# The Kinase Regulator Mob1 Acts as a Patterning Protein for *Stentor* Morphogenesis

**DOI:** 10.1371/journal.pbio.1001861

**Published:** 2014-05-13

**Authors:** Mark M. Slabodnick, J. Graham Ruby, Joshua G. Dunn, Jessica L. Feldman, Joseph L. DeRisi, Wallace F. Marshall

**Affiliations:** Department of Biochemistry and Biophysics, University of California, San Francisco, California, United States of America; Technische Universität Dresden, Germany

## Abstract

Morphogenesis and pattern formation are vital processes in any organism, whether unicellular or multicellular. But in contrast to the developmental biology of plants and animals, the principles of morphogenesis and pattern formation in single cells remain largely unknown. Although all cells develop patterns, they are most obvious in ciliates; hence, we have turned to a classical unicellular model system, the giant ciliate *Stentor coeruleus*. Here we show that the RNA interference (RNAi) machinery is conserved in *Stentor.* Using RNAi, we identify the kinase coactivator Mob1—with conserved functions in cell division and morphogenesis from plants to humans—as an asymmetrically localized patterning protein required for global patterning during development and regeneration in *Stentor*. Our studies reopen the door for *Stentor* as a model regeneration system.

## Abstract 2

Here we demonstrate that RNAi can be used in molecular studies of the giant single-celled ciliate *Stentor coeruleus*, revealing morphogenetic functions of Mob1 and highlighting the potential of this classical model for studies of morphogenesis and regeneration.

## Author Summary

Cells have the ability to develop complex morphologies, but the mechanisms that determine these varied shapes are not well understood. Cell shape determination can be challenging to study in multicellular organisms because it can be difficult to know whether shape changes are determined internally within an individual cell or externally, driven by input from neighboring cells or by both. The giant unicellular ciliate, *Stentor coeruleus*, provides an ideal single-cell model in which to study morphogenesis due to its large size and reproducible, complex patterning. Although *Stentor* was a popular experimental organism around 100 years ago, molecular tools were not subsequently developed to sustain its use as a model system today. Here we demonstrate that RNA interference (RNAi) “by feeding” is effective in *Stentor* and demonstrate its utility for studying morphogenesis and cell polarity patterning in this organism. We show that the conserved Mob1 kinase regulator protein is asymmetrically localized to the posterior end of *Stentor* and is positioned at the newly forming posterior pole during cell division, suggesting that it may have a role in morphogenesis. Using RNAi, we show that depletion of Mob1 results in *Stentor* cells with marked defects in morphogenesis. Our findings suggest that *Stentor coeruleus* can be a powerful model system studying morphogenesis and regeneration at the single-cell level and that Mob1 is a patterning protein required for its normal development and regeneration.

## Introduction

The ability to develop and regenerate complex morphologies from a simpler starting point is among the properties that set living organisms apart from inanimate matter. Although these processes are most often considered in the context of embryos and multicellular organisms, even individual cells need to develop and regenerate after injury. Metazoan development is conceptually straightforward, in that organisms rely on the existence of numerous individual cells that differentiate into various cell types with specialized functions, thereby creating the complex architecture of the larger organism. However, it is less clear how similar levels of complexity can exist in an individual cell that cannot rely on the differentiation of its subunits. The morphogenesis of individual cells represents a key process in cell and developmental biology, but its mechanisms are almost completely unknown [1],[2].

**Figure 1:**
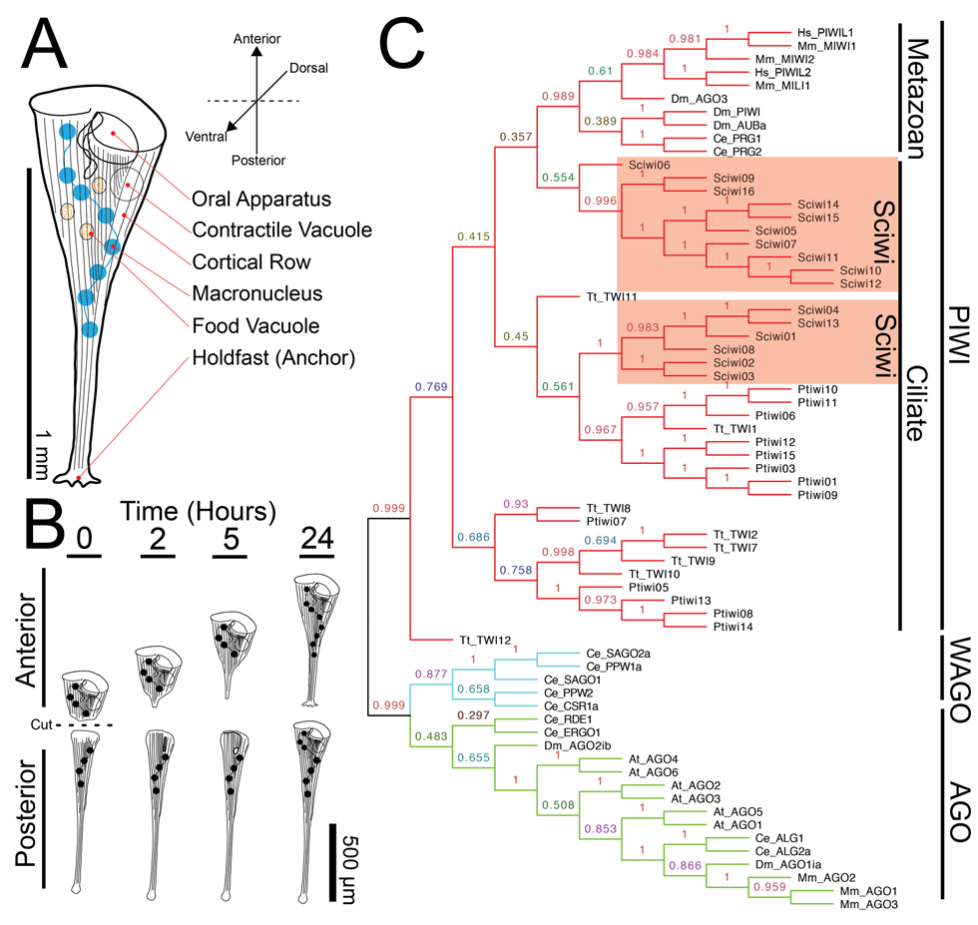
The anatomy of *Stentor coeruleus*. (A) Cartoon of *Stentor coeruleus* highlighting key cellular structures, all of which have reproducible positions within the cell. (B) Cartoon representing *Stentor* regeneration after surgical bisection as initially reported by Thomas Hunt Morgan in 1901. (C) A neighbor-joining phylogenetic analysis of protein sequences of *Stentor* argonaute homologs along with sequences from *Paramecium* (*Pt*), *Tetrahymena* (*Tt*), human (Hs), mouse (Mm), *C. elegans* (*Ce*), *Drosophila* (*Dm*), and *Arabidopsis* (*At*). The three major classes of Argonaute proteins—PIWI, WAGO, and AGO—are indicated (Sciwi proteins listed in Table S1, gene IDs in Table S2).

To understand the fundamental features of complex morphogenesis, we need a model where it can be induced in the context of a single cell. In some cases, the process of regeneration mimics that of morphogenesis, so a single-cell model for regeneration could be a very powerful tool. For this reason, we turned to the large ciliate *Stentor coeruleus* (∼1 mm long). *Stentor* was first described in 1744 by Abraham Trembley and has a long history as a classical system for studying regeneration in single cells (Figure 1A) [3]. The large size of *Stentor* cells made them amenable for surgical manipulations such as cutting and grafting, allowing experimental approaches comparable to those of experimental embryology to be applied to the study of single cells. *Stentor coeruleus*, like other ciliate organisms, is covered in cilia that are used for locomotion. *Stentor* is a filter feeder, which uses its oral apparatus (OA), a dense band of cilia around the anterior of the cell, to sweep other living cells into its mouth. It is known to feed on bacteria, algae, and even other ciliates [4]. At the posterior, *Stentor* possesses an anchoring structure known as the holdfast, or foot, which is used to transiently attach to surfaces. The OA and holdfast, along with ciliated stripes that run the length of the organism, define the cell cortex and set up the anterior–posterior, dorsal–ventral, and left–right axes, which are maintained throughout division. *Stentor* thus displays complex patterning and axiation comparable to what is seen in embryos. Perhaps the most striking property of *Stentor* is that it has the ability to regenerate an entire normal organism from only a fraction of the original cell.

Its large size, complex architecture, ease of surgical manipulations, and ability to regenerate give *Stentor* significant advantages over other ciliate models and even made it the focus of some early embryologists. Thomas Hunt Morgan showed that surgically produced cell fragments could regenerate into properly proportioned cells (Figure 1B), arguing that regeneration in *Stentor* was a strictly controlled morphological process [5]. The study of *Stentor* reached its apotheosis in the work of Vance Tartar, who made extensive use of microsurgery to understand the basic principles of morphogenesis in *Stentor* [4]. Tartar showcased the robust nature of *Stentor*'s regenerative ability in minceration experiments that disrupted the polarity of the cortex but did not prevent the cells from reestablishing normal polarity [6]. He also grafted parts of cells to one another to show that a single region of the cell known as the locus of stripe contrast could control the formation of a new body axis [7]. The ability to induce the regeneration of specific cellular structures is a major advantage of *Stentor* as a model for morphogenesis [8]–[10]. But *Stentor* was never developed as a molecular model system, and thus, despite ongoing fascination with the question of how a cell can develop such complexity, the molecular basis of pattern formation and regeneration in *Stentor* remains unknown.

Here we demonstrate that RNA interference (RNAi) technology is highly effective in *Stentor*, thus enabling us to study molecular mechanisms of *Stentor* development. For our initial attempt to use RNAi to identify a molecular determinant of morphogenesis in *Stentor*, we noted that the sequence of morphological events that take place when the cell regenerates a new OA is virtually identical to those observed when a cell forms a second OA during normal division [11]. We therefore used a candidate-based approach to delineate potential regulators of *Stentor* morphogenesis by focusing on conserved components of both cell division and polarization/morphogenesis. One potential candidate is the conserved eukaryotic kinase-regulator Mob1. Mob1p was first identified in budding yeast [12] and is part of a highly conserved family of which yeast has two members, Mob1p and Mob2p. Mob1p is involved in the mitotic exit network and is required for proper cytokinesis, whereas Mob2p is involved in the regulation of Ace2p and polarized morphogenesis network and is required for proper cell morphology [13]. Mob1 is a highly conserved kinase co-activator that binds to NDR/LATS kinases and stimulates their activity. Mob1 has been implicated in the Hippo signaling pathway in *Drosophila* [14] and plays a role in a variety of processes including apoptosis, mitosis, morphogenesis, and proliferation [15]. Recent work on the only member of the MOB family in *Tetrahymena thermophila* suggests that Mob1 function is conserved in ciliates and that Mob1 is required for proper cytokinesis, but it is unclear whether Mob1 functions in ciliate morphogenesis [16]. Here we show that Mob1 is conserved in *Stentor* and is asymmetrically localized in the cell. Using RNAi, we discovered that Mob1 is a global patterning protein that is required for proper development and regeneration.

## Results

### The *Stentor* Genome Encodes RNAi Machinery

The *Stentor* genome is currently being assembled and annotated. In order to determine if the RNAi machinery is conserved in *Stentor coeruleus* prior to completion of the *Stentor* genome, we obtained a genomic sequence using short Illumina reads that were assembled using the targeted assembly algorithm PRICE [17]. Using reads with homology to *Tetrahymena* proteins as seed sequences, we specifically assembled sequences with homology to known RNAi machinery such as Argonaute, Dicer, and RNA-dependent RNA polymerases. We were able to assemble a number of homologs for each of the RNAi machinery components (Table S1). Using a recently reported functional analysis of the Argonaute homologs in Paramecium [18] as a reference point, we performed a neighbor-joining phylogenetic analysis of *Stentor* Argonaute homologs. Like those of *Paramecium* and other ciliates, all *Stentor* Argonaute proteins cluster in the PIWI subfamily (Figure 1C); hence, we use the term Sciwi for *Stentor coeruleus* PIWI. All of the Sciwi proteins contain the conserved “DDH” motif (Figure S1), which has been shown to be necessary for the slicer activity of PIWI proteins [19].

### RNAi by Feeding in *Stentor*

**Figure 2:**
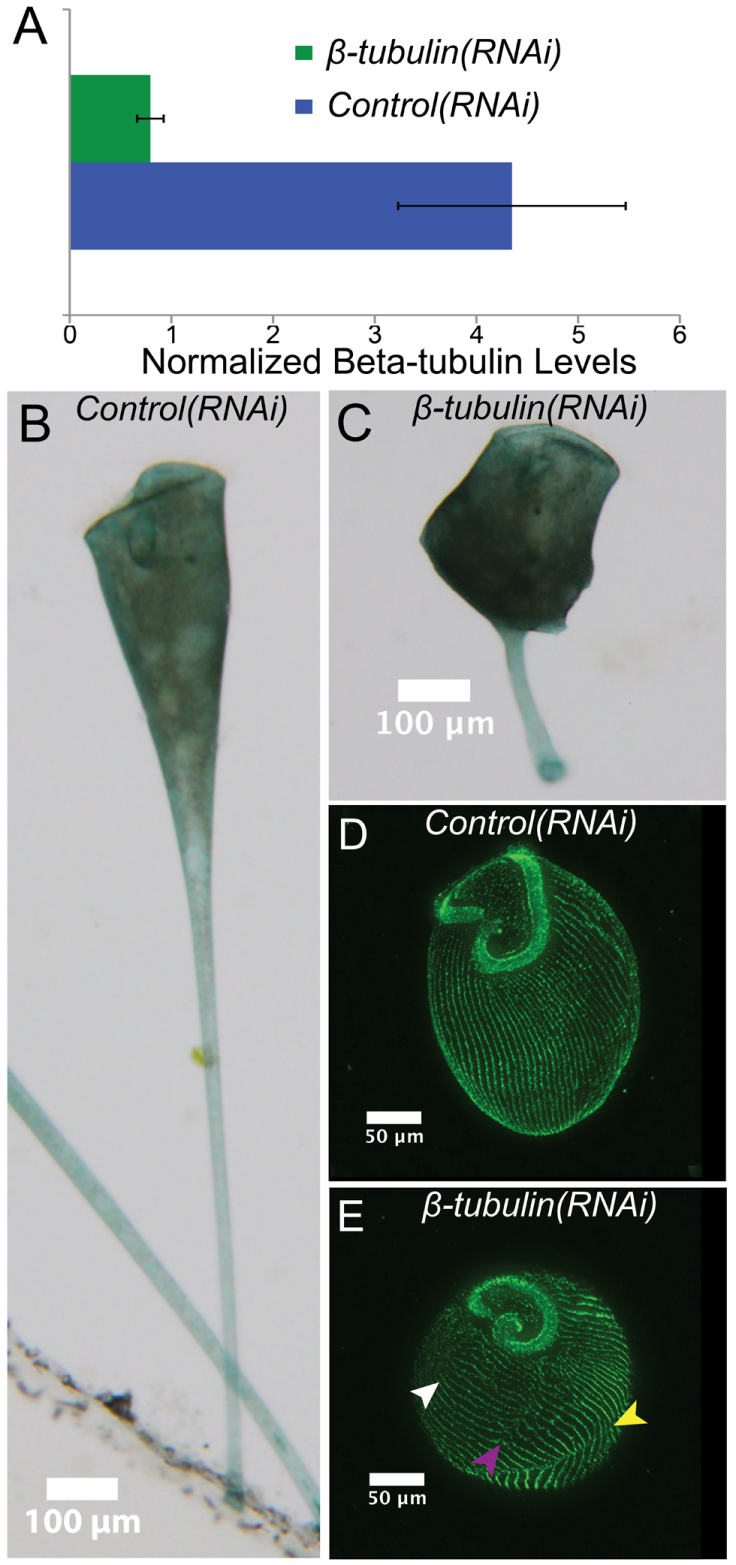
*β-tubulin(RNAi)* results in aberrant cell morphologies. (A) qRT-PCR results showing relative expression of β-tubulin normalized to GAPDH expression in both *control(RNAi)* and *β-tubulin(RNAi)* cells. (B, C) Brightfield images of control and *β-tubulin(RNAi)* cells showing dramatic alteration in cell shape. (D, E) Immunofluorescence images of stained control and *β-tubulin(RNAi)* cells highlighting cortical microtubule bundles (green, anti-α-tubulin). In contrast to the highly organized parallel microtubule rows seen in control cells, *β-tubulin(RNAi)* cells have improperly oriented (white arrow), broken (purple arrow), and discontinuous (yellow arrow) rows, indicating a disruption of normal cellular patterning.

Based on the high sequence conservation of the RNAi machinery, we asked whether gene expression could be perturbed by RNAi in *Stentor*. RNAi has been performed in two other ciliates, *Paramecium tetraurelia* and *Blepharisma japonicum*, using the method of feeding with bacteria expressing double-stranded RNA [20],[21]. However, in other ciliates, such as *Tetrahymena thermophila*, RNAi by feeding does not work. To test whether RNAi by feeding is effective in *Stentor*, we performed a knockdown of α- and β-tubulin—key components of the cortical structures in *Stentor*—by feeding bacteria containing an expression plasmid encoding dsRNA directed against α- or β-tubulin. There were eight α-tubulin and six β-tubulin homologs identified from the PRICE assembly and at least one shared ≥20 mer among all of the sequences. We hypothesized that since tubulin is a key component of the cell structure, its knockdown would display a clear phenotype as a proof of principle for RNAi. We found that RNAi resulted in a significant knockdown at the level of the transcript (Figure 2A). Targeting either α- or β-tubulin with RNAi vectors caused cells to take on a rounded shape not seen in untreated cells after 5 d of feeding (Figures 2B,C and S2). Identical results were obtained targeting either tubulin gene or either half of the tubulin genes individually, arguing the result was not an off-target effect (Figure S2C). Using antibodies against α-tubulin to highlight the cortical rows, we observed that tubulin knockdown resulted in the disorganization of cortical rows (Figure 2D,E). We also noted that the macronucleus in tubulin knockdown cells often collapsed into two large nodes, one located near the anterior and one at the posterior pole of the cell (Figure S2B). This failure to maintain an elongated macronucleus is consistent with a previous observation that microtubules are involved with elongation [22]. Cells depleted of tubulin appear to sustain cortical damage such as breaks and discontinuities of the cortical rows, which they are unable to repair properly (Figure 2E, arrows). Tartar found that cortical discontinuities induced by surgery often resulted in transient protrusions, resembling posterior poles, extending from the cell [6]. Consistent with that observation, we found that some tubulin knockdown cells formed ectopic posterior poles, suggesting a role for an organized cortex in the maintenance of cell polarity (Figure S2D).

To demonstrate that the morphological defects seen in Figure 2 are specific for tubulin RNAi, we performed RNAi using a gene whose function is predicted to be unrelated to cortical row organization, namely the ciliary length regulating kinase LF4 [23],[24]. When LF4 was knocked down via RNAi in *Stentor*, the cilia increased in length, but tubulin staining of cortical rows as well as cell shape and patterning were unaffected (Figures 2D and S3A–D). This result rules out the possibility that activation of the RNAi machinery causes nonspecific changes in cell morphology. Additionally, RNAi using sequences targeted to planarian ODF2 and unc22, genes not present in ciliates, resulted in normally shaped cells (Figure S3E,F). These data show that RNAi constitutes a powerful tool for studying the molecular mechanisms of regeneration and morphogenesis in *Stentor*.

### Mob1 Localization in *Stentor*

**Figure 3:**
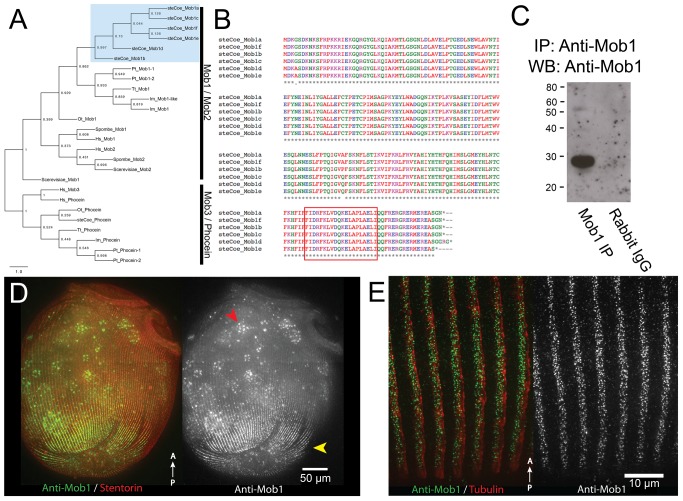
Conservation of Mob1 and its localization in *Stentor.* (A) Maximum-likelihood phylogenetic tree showing *Stentor* Mob1's position relative to other MOB family sequences from Human (Hs), *S. cerevisiae*, *S. pombe* (Sp), Tetrahymena (Tt), Paramecium (Pt), Ichthyophthirius (Im), and Oxytricha (Ot). Cluster of *Stentor* sequences is indicated with a blue box. (B) Alignment of *Stentor coeruleus* Mob1 protein sequences. The peptide sequence used to generate the Mob1 antibody is indicated by the boxed region near the C terminus. (C) IP:Western blot showing the Mob1 band present at 26 kD. Rabbit IgG alone was run to gauge the level of background signal generated by the secondary antibody to rule out the contaminating signal from IgG light-chain, which is around the same size as Mob1. (D) Immunofluorescence image of cells showing Mob1 localization (green, anti-Mob1) and cortical rows (red, stentorin-autofluorescence). Mob1 localizes to the OA in the anterior and cortical rows of the posterior (yellow arrow). There is background signal from autofluorescent food vacuoles containing *Chlamydomonas* (red arrow). (E) Immunofluorescence image showing a high magnification view of Mob1 localization (green, anti-Mob1) at the posterior, which is punctate and diffusely labels the cortical rows marked by the tubulin staining (red, anti-acetylated tubulin).

Having established the efficacy of RNAi, we set out to use this method to test the function of a candidate morphological determinant, Mob1, based on the reasoning outlined in the introduction. From the targeted PRICE assembly, we discovered a total of six genes with high homology to Mob1 (Figure 3A). A seventh sequence was identified with homology to Phocein, a protein that shares the MOB/Phocein domain that defines the family (Figure 3A). All six putative Mob1 homologs were 99% identical to each other at the protein level (Figure 3B) and shared 52% identity with Mob1 versus only 38% identity with Mob2 protein sequences from *S. pombe* and we refer to them as Mob1. To determine the localization of Mob1 in *Stentor*, we generated a polyclonal antibody against a *Stentor* Mob1 peptide sequence shared between all six identified proteins. On Western blots of Mob1 immunoprecipitated from *Stentor* lysates, the affinity-purified *Stentor* Mob1 antibody recognized a single band of the appropriate size at 26 kDa (Figure 3C). When used for immunofluorescence, the antibody clearly labeled the posterior and appeared to label the region around the OA, although this staining was less clear (Figure 3D). This localization pattern was blocked by pre-incubating the primary antibody with the immunizing peptide, which suggested that it is specific to the Mob1 family and not the result of a nonspecific antibody binding (Figure S4). This dual localization pattern was similar to the pattern seen in *Tetrahymena* [16]. Interestingly, unlike in *Tetrahymena*, the antibody did not appear to exclusively label basal bodies in *Stentor*, but rather diffusely labeled the cortical rows (Figure 3E).

**Figure 4:**
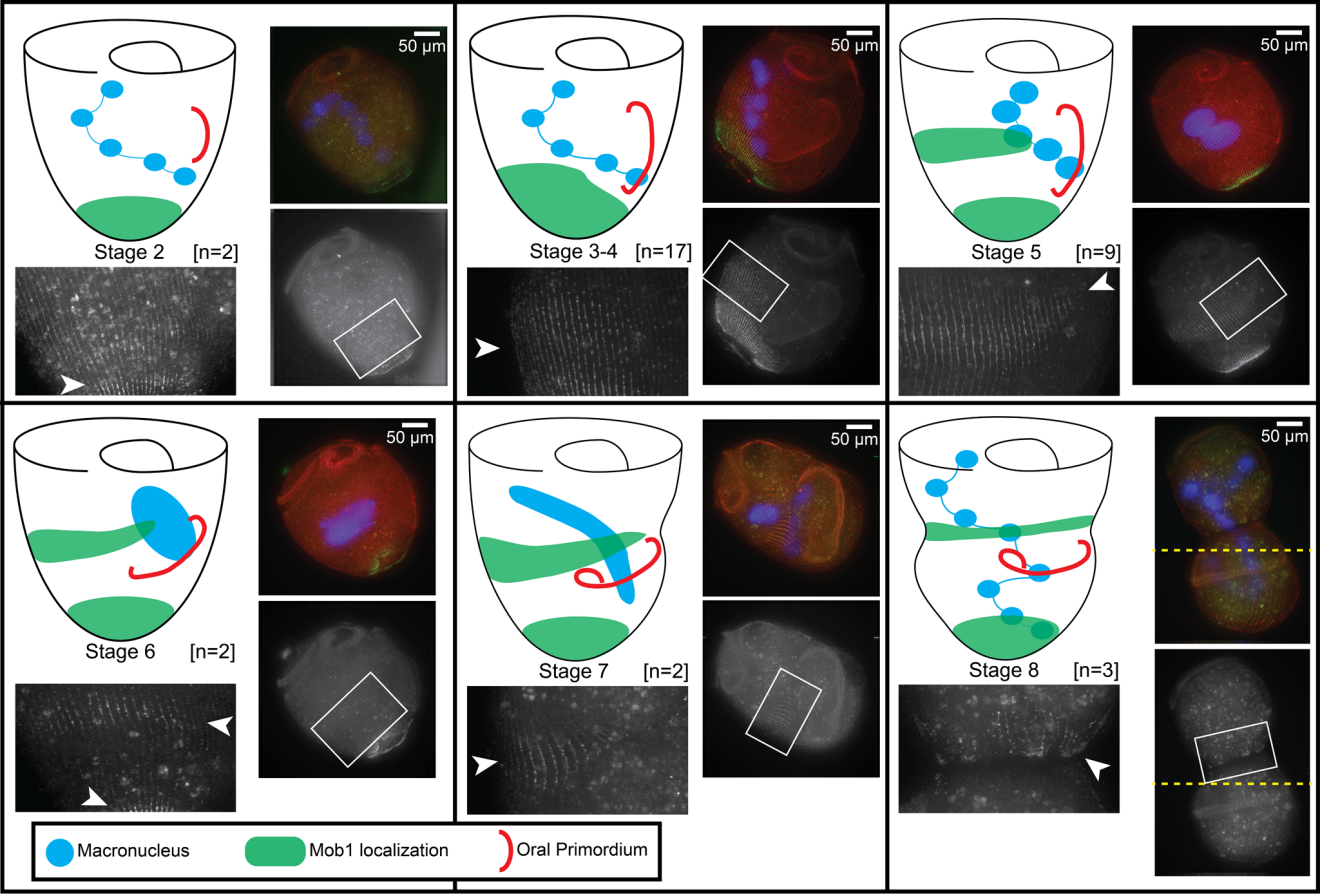
Mob1 localizes to the presumptive posterior during cell division. Cartoon showing Mob1 localization next to representative immunofluorescence images for each stage of division. Regions indicated by the white boxes are magnified in the adjacent images to clearly show Mob1 localization. Number of cells observed at each stage: stage 2 (*n* = 2), stages 3–4 (*n* = 17), stage 5 (*n* = 9), stage 6 (*n* = 2), stage 7 (*n* = 2), and stage 8 (*n* = 3) are represented, with staining in all cells consistent with the diagram. Mob1 appears to migrate up from the posterior during stages 3–4 and focuses into a band around the midline in stage 5. Through the end of division, this band is maintained and forms a clear border between the two daughter cells, which later becomes the posterior end of the anterior daughter cell. High levels of background autofluorescence from *Chlamydomonas* containing food vacuoles is often a problem because cells were fed continuously for this experiment, but Mob1 staining can be seen specifically along cortical rows indicated by white arrowheads. The cells imaged in stage 7 were too large to fit in a single frame, and two separate images were taken with identical settings, then manually stitched together using the cortical rows for alignment (join-point of the two images is indicated by a dashed yellow line).

To get a better idea of Mob1 localization throughout the cell cycle, we followed dividing cells and fixed them at different stages of division. Division proceeds through a series of eight morphologically defined stages [4]. During stage 1 the oral primordium begins to form as a clearing of rows along the locus of stripe contrast at the midline of the cell, which expands during stage 2. In stages 3 and 4, this clearing is filled by the synthesis of new basal bodies, which are then ciliated as the oral primordium increases in length. In stage 5, the cell elongates as the oral primordium further develops and the macronucleus begins to condense. By stage 6, the macronucleus collapses into a single large node, and cortical partitions between the anterior and posterior daughter cells become visible. Finally, during stages 7 and 8, the macronucleus extends back to its normal shape and is divided between the two daughters as the oral primordium is positioned at the presumptive anterior of the posterior daughter and the posterior of the anterior daughter is constricted to form a new holdfast and the cells are finally separated. Because there are no described methods to synchronize *Stentor* cells, we observed vegetatively growing cultures and isolated cells that presented visible evidence of cell division. Although the earliest stages of division are difficult to identify within a culture, we were able to isolate cells from stage 2 all the way through stage 8 (Figure 4). From these data, we were able to determine that Mob1 expands its posterior localization by stage 3 or 4. By stage 5 that expansion begins to focus into a discrete band around the midline, and by stage 6 this band spread around the cell, anterior of the oral primordium and is positioned near the presumptive posterior pole of the anterior daughter cell. During stages 7 and 8, this band clearly defined the constriction of the newly forming posterior and there was a clear break between the two halves of the dividing cell. Thus, Mob1 appears to localize at the posterior end of the anterior daughter cell prior to completion of cell division.

### Mob1 RNAi Results in Defective Morphogenesis

**Figure 5:**
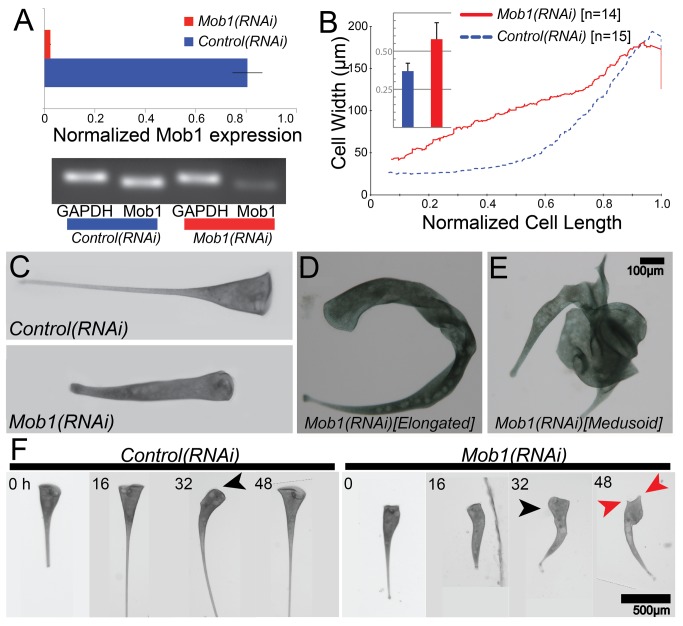
*Mob1(RNAi)* cells lose proper cell proportions and body axes. (A) qRT-PCR data showing relative expression of Mob1 normalized to GAPDH expression in control and *Mob1(RNAi)* cells. (B) Quantitative analysis of the proportionality defect plotted as a graph of cell width versus normalized cell length for *control(RNAi)* (blue, *n* = 14) and *Mob1(RNAi)* (red, *n* = 15) cells. Data for each line represent a moving average of all samples with a window size of 2*n*. These measurements were used to compute a shape factor as described in Materials and Methods and graphed in the inset bar graph. The shape factor describes deviation from a shape having perfect straight lines on the cell edge. Data in the inset show an increase in shape factor from 0.37±0.052 (*n* = 15) in control cells to 0.58±0.108 (*n* = 14) in *Mob1(RNAi)* cells, a highly significant increase (*p* = 1.8×10^−6^). (C) Control cell's canonical shape as compared to a *Mob1(RNAi)* cell fed the RNAi vector for 3 d, showing altered cell proportionality. (D, E) Brightfield image of an elongated (D) and medusoid (E) cell. (F) Selected images from a time course. *Control(RNAi)* and *Mob1(RNAi)* cells were isolated after 2 d of feeding and imaged every 2 h for an additional 52 h. Spontaneous reorganization of the OA (black arrows) occurred prior to the multipolar phenotype (red arrows) in *Mob1(RNAi)* cells.

To determine the function of Mob1 in *Stentor* cells, we created an RNAi vector targeting Mob1 sequence. Because of the high sequence similarity among the *Stentor* Mob1 homologs, 85%–95% identity at the nucleotide level (Figure S5), we expect that any long dsRNA Mob1 construct would target all six Mob1 genes, although we specifically used Mob1a for this study. When aligned pairwise with all other *Stentor* Mob1 homologs, Mob1a shared at least one ≥20 mer between the sequences for all possible pairs and so it is possible that this single construct would be sufficient for the knockdown of all Mob1 genes. Additionally, RNAi constructs were made specifically targeting Mob1b, c, and d as well and all gave identical results (unpublished data). Because the MOB family of proteins has conserved functions in both cell division and morphogenesis, we expected phenotypes that would affect cytokinesis and cell polarity [15]. RNAi knockdown of Mob1 in *Stentor* was extremely effective and resulted in a 30-fold reduction of Mob1a transcript levels compared to the GAPDH control after 4 d of feeding (Figure 5A). This treatment caused dramatic defects in *Stentor* morphology, which progressively worsened as feeding of the RNAi vector continued. After 24–48 h of RNAi, we observed cells with altered cell proportionality; cells had lost their characteristic “wine-glass” shape and became more cylindrical (Figure 5B,C and S6). Mob1 thus appears to play a key role in the regulation of proportional cell shape, the phenomenon first characterized by Morgan in his landmark 1901 paper [5]. Between 48 and 96 h of Mob1 knockdown, cells displayed further morphological abnormalities that could be separated into two categories. The first consisted of cells that were highly elongated and curved, apparently a result of a deformed cortex, which caused the cells to twist (Figure 5D). The other class of defects consisted of multipolar (medusoid) cells with multiple OA regeneration bands and ectopic tails, growing from the cell body, that were often functional posteriors (Figure 5E). These morphological effects were not observed with RNAi targeting any other genes we tested, suggesting they are specific to the Mob1 knockdown. Identical phenotypes were observed when either half of the gene was targeted separately (Figure S7 and Movies S1–S3). In addition to these morphological defects, Mob1 knockdown cells show clear defects in cytokinesis (Figure S8), comparable to those observed in *Tetrahymena*, although cell division was rare and seen in less than 5% of *Mob1(RNAi)* cells over a 5-d period, which is typical for *Stentor* in our growth conditions [16].

Some of the more severely affected medusoid cells were so abnormally shaped that it was impossible to define what had happened to the cells from only a single time-point, raising the possibility that multiple failed attempts at cell division might have played a role in development of the phenotype. To obtain a clear idea of the development of these phenotypes, we imaged individual cells every 2 h after feeding them the RNAi vector for 48 h. We observed that all cells went through similar stages of aberrant morphogenesis (Figures 5F and S9), initially losing their canonical wine-glass proportions and elongating slightly relatively early in the time course (Figure 5F, 16 h), and eventually converting to the medusoid form. During the evolution of the *Mob1(RNAi)* phenotype, cells underwent a round of spontaneous regeneration of the OA. This is a normal process in *Stentor* and does not normally result in aberrant morphogenesis, but in *Mob1(RNAi)* cells, spontaneous OA regeneration was immediately followed by off-axis growth—that is, the extension of a new posterior pole along an axis different from the previously existing anterior–posterior axis, indicating that this process might trigger the development of further defects (Figure 5F, 32 h). The cell cycle of *Stentor* is between 96 and 120 h in our growth conditions, and consistent with this long duration, we found that no cells initiated cell division during the 52-h observation period, making it unlikely that the observed morphological defects could be products of failed cytokineses (*n* = 20). These data suggest that Mob1 is required for OA localization and for the proper regulation of posterior structures; and in the absence of Mob1, posterior growth becomes unregulated. However, our results also imply that regeneration of the OA might be triggering the development of more severe defects and a switch from disproportioned and elongated bipolar cells to multipolar cells.

**Figure 6:**
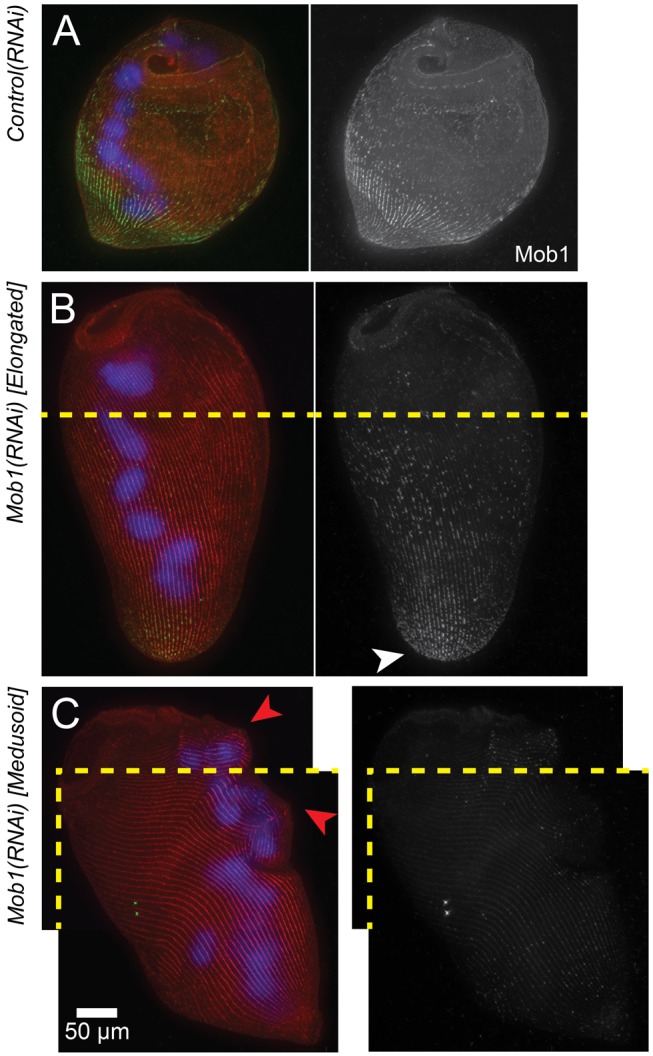
Presence of residual Mob1 protein in *Mob1(RNAi)* cells. (A) Immunofluorescence images of stained *control(RNAi)* cells showing Mob1 localization (green, anti-Mob1), macronucleus (blue, DAPI), and cortical rows (red, stentorin-autofluorescence). (B, C) Immunofluorescence images of stained elongated (B) and medusoid (C) *Mob1(RNAi)* cells; Mob1 (green, anti-Mob1), macronucleus (blue, DAPI), and cortical rows (red, stentorin autofluorescence) (compare to A). Cortical aberrations are seen in the cortical rows (red arrows). Both the elongated and medusoid cells were too large to fit in a single frame, and two separate images were taken with identical settings, then manually stitched together using the cortical rows for alignment (join-lines between images are indicated by a dashed yellow line).

Interestingly, when we localized residual Mob1 protein at different stages in *Mob1(RNAi)* cells (Figure 6), we noted that Mob1 protein is first lost from more anterior regions (Figure 6B), and only by the medusoid stage is Mob1 staining almost completely absent (Figure 6C). This raises the possibility that differentially localized Mob1 is performing different functions in *Stentor*, and its loss in these specific locations triggers the development of different phenotypes.

### Mob1 Function in Regeneration

**Figure 7:**
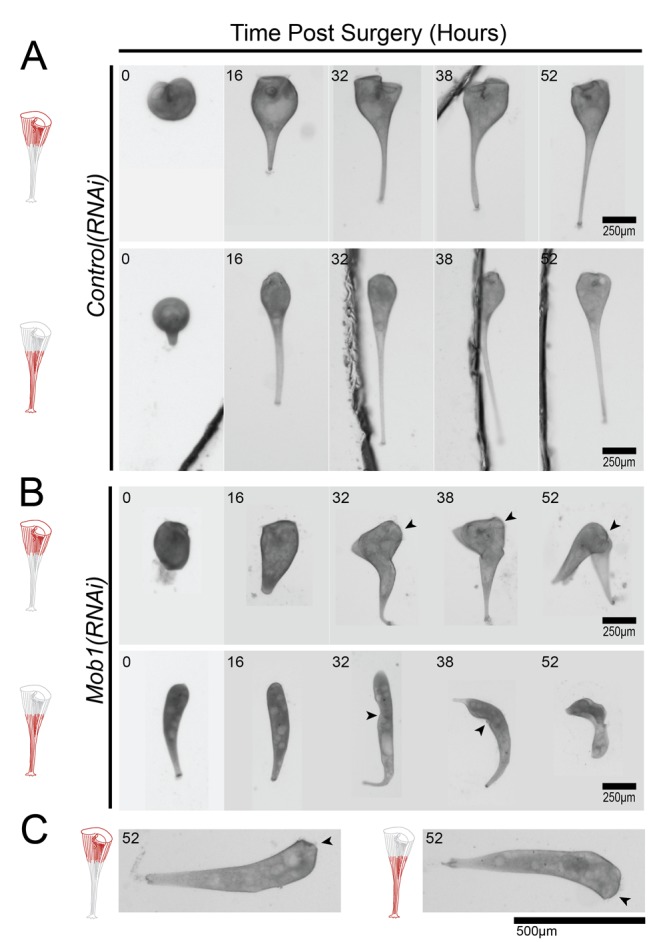
Morgan revisited: regeneration of proportionate structures in *Stentor* requires Mob1 protein. (A–C) Observed fragments of cells are shown in red, and OAs are indicated with black arrowheads where they are difficult to identify. (A) Regeneration of both anterior and posterior fragments of control cells after surgical bisection. (B) Regeneration of both anterior and posterior fragments of bisected *Mob1(RNAi)* cells after 3 d of feeding on the RNAi vector. (C) Anterior and posterior *Mob1(RNAi)* cell fragments regenerated OA/holdfast properly in 10% of cells but failed to reestablish normal cell proportions (*n* = 20).

We next hypothesized that if different populations of Mob1 perform different functions in the cell, we would be able to determine these functional differences using microsurgery to remove specific regions of the cell containing Mob1. In the case of a simple bisection, the anterior fragment of the cell would lack the posterior population of Mob1 and need to regenerate posterior structures, whereas the posterior fragment would lack the anterior population of Mob1 and need to regenerate a new OA and anterior (Figure 1B). Morphologically normal cells, taken after 72 h of feeding the Mob1 RNAi vector, were bisected and those fragments were observed every 2 h. Compared to control cells (Figure 7A), *Mob1(RNAi)* cell fragments grow ectopic tails resembling normal posterior structures (Figure 7B). Anterior fragments maintained the original OA and only grew ectopic tails adjacent to the previous posterior structures, which would suggest that the OA has some control over posterior growth. Conversely, posterior fragments failed to properly localize their regenerating OA, which remained on the dorsal side of the cell, and resulted in cells that were able to grow new posterior structures at the anterior end. These results show that Mob1 is not required to initiate regeneration, although once initiated neither the anterior nor posterior halves properly regenerated the OA or the holdfast. Furthermore, this suggests that Mob1 plays a key role in defining polarity and regulating polarized cell growth during normal development as well as regeneration.

Interestingly, 10% of cells were only mildly affected and successfully regenerated their missing structures (holdfast and OA). However, they still lost normal cell proportions, indicating that the RNAi had occurred in these cells (*n* = 20, Figure 7C). We hypothesize that these cells represent incomplete knockdown of Mob1 and that cell proportionality is more sensitive to Mob1 depletion than OA and posterior pole formation. The fact that proportionality defects can occur without inducing regeneration suggests that these two phenotypes are functionally separable.

**Figure 8:**
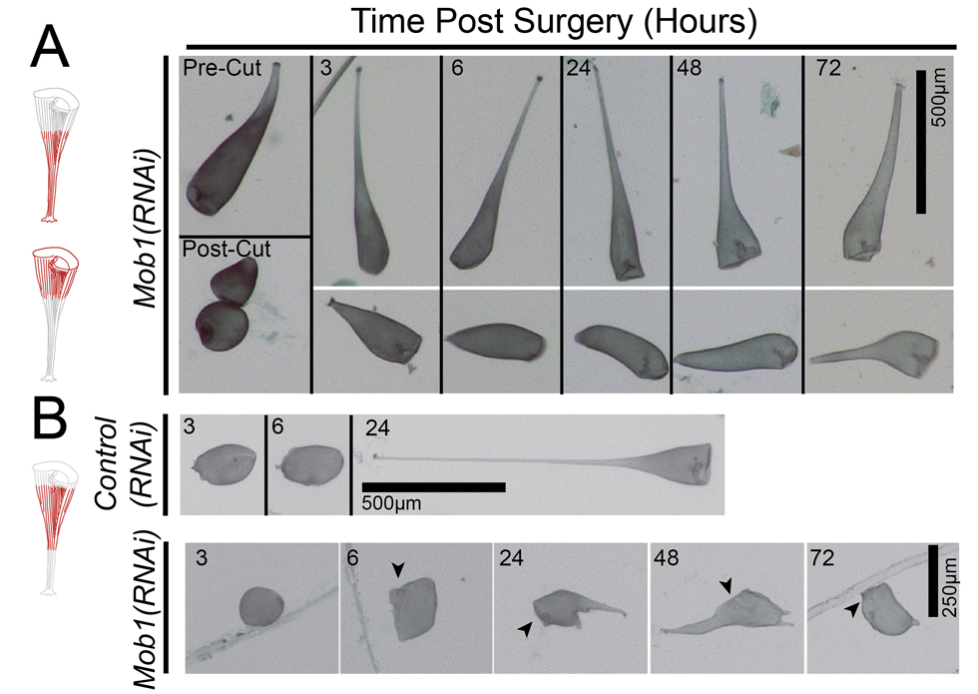
Residual Mob1 in the anterior and posterior can be surgically removed. (A, B) Observed fragments of cells are shown in red, and OAs are indicated with black arrowheads where they are difficult to identify. (A) Mob1(RNAi) cells that are bisected only 2 d after feeding the RNAi vector are capable of normal regeneration. (B) *Control(RNAi)* and *Mob1(RNAi)* cells had their anterior and posterior regions excised to remove regions of the cell where Mob1 localizes. Only removal of both Mob1-containing poles in *Mob1(RNAi)* cells prevented regeneration at this early stage of the RNAi time course.

A challenge for using RNAi to study development is that phenotypes can take time to fully develop because protein turnover takes a longer time than transcript knockdown. Such a lag between message depletion and protein depletion is a universal feature of RNAi in all organisms and simply reflects the greater stability of protein compared to mRNA. In the case of *Stentor*, Mob1 knockdown cells observed 48 h into the RNAi time course still showed normal morphology and were able to fully regenerate after bisection, to an extent comparable to *control(RNAi)* cells (Figure 8A), despite the fact that mRNA levels were dramatically reduced relative to controls. This phenotypic lag relative to the timing of mRNA knockdown along with immunofluorescence data that clearly show the presence of Mob1 protein in the posterior even in elongated cells (Figure 6B) suggested that there could still be a sufficient amount of Mob1 protein to function during regeneration. In most systems, there is no way to bypass this phenotypic lag and one must simply accept it as a caveat for RNAi experiments, but in our case the ease of *Stentor* manipulation provides a way to speed up the development of an RNAi phenotype by physically removing the parts of a cell where the target protein resides. To this end, we surgically removed the head and the tail, which are the portions of the cell where the majority of Mob1 protein is localized, after inducing Mob1 knockdown by RNAi. If the phenotypic lag was due to retained protein in these regions, this surgical operation should reduce the lag between mRNA knockdown and development of morphological phenotypes. In *Control(RNAi)* cells, removal of both the head and tail structures yielded morphologically normal cells after 24 h (Figure 8B, top), with Mob1 signal returning as early as 3 h postsurgery as observed by immunofluorescence (Figure S10). However, when both the heads and tails were removed from morphologically normal *Mob1(RNAi)* cells at an early stage of knockdown when cells still showed normal morphology, they developed phenotypes similar to those seen at much later stages of Mob1 knockdown (Figure 8B, bottom). The result that surgically accelerated removal of Mob1 proteins reduces the lag between gene knockdown and development of morphological phenotypes supports the idea that Mob1 protein functions globally in establishing both anterior and posterior polarity in *Stentor*.

## Discussion

The ability to perform RNAi in *Stentor* to manipulate genes of interest, such as we have done with Mob1, will pave the way for many future studies to unravel the mechanism of single-celled pattern formation and regeneration. Although the standard drawbacks of RNAi still apply to *Stentor*—namely, the cell-to-cell variability in the level of knockdown and phenotypic lag due to target protein stability—*Stentor* provides unique methods for addressing these issues because manipulating individual cells is trivial and surgical removal of the protein pool is possible, at least when the target protein is concentrated in a specific region of the cell.

These results, to our knowledge, represent the first molecular analysis of regeneration in *Stentor* to be reported and build on observations of proportional regeneration first made by Morgan over 100 years ago. The kinase co-activator Mob1 is clearly localized to the posterior in vegetative cells. At distinct stages during cell division, Mob1 is found to first expand toward the anterior, where it is later focused into a discrete band around the midline of the cell. Toward the end of division, it creates a clear boundary between the daughter cells, where it localizes to the newly forming posterior of the anterior daughter cell. Localization of Mob1 to the midline of dividing cells is not unique to *Stentor* and is comparable to observations of Mob1 in a variety of other organisms, including *Tetrahymena* [16], Alfalfa [25], and budding yeast [26], although it is interesting to note that Mob1 is clearly asymmetrically localized to the anterior daughter at the midline of both *Stentor* and *Tetrahymena* during division.

Loss of Mob1 due to RNAi knockdown results in a loss of normal proportions, apparent uncontrolled cell growth, and cytokinesis defects. When considering these data alongside the data from *Tetrahymena*, it certainly suggests that the single ciliate MOB family member might share the more specialized functions of the multiple MOB family members in other organisms, which has also been suggested by Tavares et al. [16]. Although it is still unknown if any of the functional interactions of the MOB family are also conserved in ciliates, such as specific interactions with NDR kinases and STE-like kinases, we hope to address these questions in the future with the advent of a more complete *Stentor* genome.

From these data we can conclude that Mob1 is essential for maintenance and regeneration of cell polarity and proper cell proportions. We also show that RNAi by feeding can now be used as a routine tool to study morphogenesis and regeneration at the level of single cells in *Stentor*. There are likely to be many localized pattern regulatory proteins in addition to Mob1 that control development in *Stentor*, and Mob1 will serve as a model for their study. With this remarkable single-cell system, we have opened the doors to studying the molecular mechanisms of regeneration at a resolution impossible to attain in other regenerative models. Moving forward we hope to develop more ways to manipulate *Stentor* and further investigate the role of Mob1, and its associated pathways, in order to expand our knowledge of cell polarity, regeneration, and morphogenesis.

## Materials and Methods

### Culturing and Media

*Stentor coeruleus* cells were obtained commercially (Carolina Biological Supply, Burlington, NC) but subsequently maintained in culture within the lab by growing in the dark at 20 °C in Modified *Stentor* Medium (MSM), 0.75 mM Na_2_CO_3_, 0.15 mM KHCO_3_, 0.15 mM NaNO_3_, 0.15 mM KH_2_PO_4_, 0.15 mM MgSO_4_, 0.5 mM CaCl_2_, and 1.47 mM NaCl modified from the original recipes described by Tartar [4] and De Terra [27]. This medium provides no nutrients and must be supplemented with living prey. In order to provide prey with a known genome, we use *Chlamydomonas reinhardtii* grown separately in TAP medium [28] and washed in MSM before feeding. The 300 mL *Stentor* cultures are given 3×10^7^ *Chlamydomonas* cells two or three times per week.

### Cloning of *Stentor* Gene Sequences

Homologs were identified by best-reciprocal BLAST starting with *Paramecium tetraurelia* proteins (Table S1). Target gene sequences were obtained by PCR amplification from genomic DNA and cloned into pPR-T4P (kind gift from J. Rink), a modified pDONR-dT7 in which a ligation-independent cloning site was added [29]. Cloning was performed by either the ligation-independent method or cohesive-end ligation. Additional information about the RNAi constructs used in this study is included in Table S3.

### Phylogenetic Analysis

Multiple sequence alignments were made using ClustalW2 with default settings. The list of Argonaute proteins used in the analysis is included in Table S2. The un-rooted neighbor-joining tree was made with 1,000 bootstrap replicates using the MEGA v5.1 program [30]. FigTree v1.4 was used to visualize the tree data.

### RNAi

RNAi was performed by transforming HT115 *E. coli* with each plasmid to allow for dsRNA expression of the target gene. Transformed bacteria were grown to log phase and then induced with 1 mM IPTG for 3 h at 37 °C. After induction, bacteria were washed and resuspended in MSM, then fed to *Stentor* that had been previously starved for 24–48 h. Induction and feeding of bacteria was then repeated for 2–5 d. Negative controls used for RNAi experiments were either pPR-Sciwi03 or pPR-LF4.

### Quantitative PCR Assay

RNA was extracted from 50 cells per sample using PureLink RNA mini kit (Life Technologies, Grand Island, NY). After purification, RNA was treated with DNaseI (New England Biolabs, Ipswitch, MA), repurified, and then primed with oligo-dT and reverse transcribed using the SuperScript III kit (Life Technologies, Grand Island, NY). Samples were diluted as necessary, and 5 µL were used in each qRT-PCR reaction. Reactions were run on a C1000 ThermoCycler (Bio-Rad, Hercules, CA) with an annealing temperature of 54 °C. Primer sets were designed for α- and β- tubulin, GAPDH, and Mob1 (Table S4). Each qRT-PCR run was finished with a melt curve to determine the homogeneity of the amplified product. Starting quantity was calculated using a standard curve and a genomic DNA control for each primer pair. Three technical replicates were performed for each of 1–3 biological replicates. Error bars represent standard deviation for biological replicates. For samples with one biological replicate, standard deviation of technical replicates is shown with uncapped error bars.

### Antibodies

Mouse monoclonal anti-acetylated tubulin (clone 6-11B-1) was used at a 1∶500 dilution (Sigma, St. Louis, MO). MOB1 antibody was generated in rabbits whose pre-immune bleeds had been screened before immunization using the synthetic peptide N-CFIDRFKLVDQKELAPLAELI-C (Covance, Denver, PA) and affinity purified using a SulfoLink Immobilization Kit for Peptides (Pierce Biotechnology, Rockford, IL). Purified Mob1 antibody was used at a concentration of 3 µg/mL. Alexa-488 goat–anti-mouse and Alexa-488 goat–anti-rabbit secondary antibodies (Life Technologies, Grand Island, NY) were used for immunofluorescence, and IRDye 800CW goat–anti-mouse and IRDye 680RD goat–anti-rabbit secondary antibodies (LI-COR Biosciences, Lincoln, NE) were used for Western blotting.

### Immunofluorescence

Cells were isolated from culture and washed in fresh MSM. Cells were then isolated in minimal volume in a 1.5 mL tube for fixation and staining in suspension. Cells were fixed in ice-cold methanol for 10 min at −20 °C, rehydrated at room temperature in a 1∶1 MeOH:PBS mixture for 5 min, and 1× PBS for 10 min. Cells were blocked in 1× PBS, 2% BSA, and 0.1% Triton-X-100 for 1 h at room temperature. In order to avoid centrifugation, cells were allowed to settle to the bottom of the tube between steps.

### Immunoprecipitation/Western Blotting

A total of 1,500 *Stentor* cells were washed 3× in MSM and 1× in ice-cold MSM and lysed in 50 mM Tris-HCl pH 8.0, 125 mM NaCl, 1% NP-40 containing complete protease inhibitor cocktail tablets (Roche Diagnostics Corp., Indianapolis, IN), mixed by pipetting and incubated for 30 min while rotating at 4 °C. Lysates were centrifuged at 10,000×*g* for 15 min at 4 °C, and the supernatant was incubated with anti-Mob1 antibody for 2 h while mixing at 4 °C. Samples were then incubated with Protein A Anti-Rabbit IgG beads overnight while mixing at 4 °C (Rockland Immunochemicals Inc., Boyertown, PA). Sample buffer was added and boiled for 10 min before running on a 10% SDS-PAGE gel and transferred onto a nitrocellulose membrane. Blots were probed with anti-Mob1 primary antibody (1∶500) and Rabbit IgG TrueBlot secondary antibody (1∶1,000) (Rockland Immunochemicals Inc., Boyertown, PA), developed using Chemiluminescent HRP substrate, and exposed to film.

### Imaging

Brightfield images were collected on a Stemi 2000C and an Axio Zoom V16 equipped with a 1× and 2.3× objective (Carl Zeiss MicroImaging, Thornwood, NY). Images were captured using an AxioCam MRc digital microscope camera (Carl Zeiss MicroImaging, Thornwood, NY) or a Rebel T3i digital SLR camera (Canon U.S.A., Inc., Melville, NY). DIC images were captured on an Axiovert 200M microscope (Carl Zeiss MicroImaging, Thornwood, NY) equipped with 10× 0.22 NA and 40×0.75 NA objectives with an AxioCam MRm digital microscope camera (Carl Zeiss MicroImaging, Thornwood, NY). Fluorescence images were collected on a Deltavision deconvolution microscope (Applied Precision, Issaquah, WA) equipped with 10×0.4 NA, 20×0.5 NA, and 100× oil 1.4 NA objectives using a CoolSnap HQ (Photometrics, Tucson, AZ) digital microscope camera. Immunofluorescence images are Z-stacks taken with 2 µm step sizes for 20× images and 0.2 µm step sizes for 100× images. Images of cells that were too large to fit into a single image were manually stitched using the cortical rows to align the two images, and the seam is indicated with a yellow dashed line.

### Cell Shape Analysis

Brightfield images of live, fully extended cells were first binarized using ImageJ v1.46. A custom MATLAB program was then used to create an outline and midline of the binarized cell image (Figure S3; code available upon request from the authors). Perpendicular lines were computed every 10 pixels along the length of the midline and their intersections with the cell outline calculated to define the cell width. Cell lengths were then normalized, and cell width versus cell length was plotted for all cells. The plot shown is a trendline of data from all cells, with a sliding average of 2*n* the number of samples collected.

In order to compare cell shapes between control and RNAi cells using this analysis, we define a shape factor that summarizes the shape of each cell as follows: for each cell, the widest point of the cell outline is assumed to represent the OA, whereas the point farthest away from the OA is assumed to represent the tail. The area of the cell contained between those two extremes is then calculated by numerical integration using the trapezoidal rule. For comparison, the area of the right trapezoid was constructed by drawing a straight line from the tail point to the OA outline point. We then define the shape factor as the ratio of the actual area to the area of the right trapezoid. This unit-less parameter will have a value of 1 if the cell is a perfect right cone—that is, if its sides are perfectly straight (a cylinder would be a special case of this). Thus, the more conical or cylindrical the cell is, the closer the shape factor gets to 1. If the cell has a taper like a wine glass or a wild-type *Stentor* cell, it will have a shape factor of less than 1. The change in cell shape from tapering to cylindrical seen in Mob1 RNAi is thus reflected by an increase in shape factor.

### Microsurgery

Surgery was performed following methods reported by Tartar [7]. Cells were isolated from culture and washed in fresh MSM. Cells were transferred to 1–2% Methylcellulose (Sigma, St. Louis, MO) in MSM, mounted on a slide in a 1 cm × 1 cm well, and visualized on a Olympus Stemi-2000c stereoscope. Microsurgery was performed using glass-stirring rods (Fisher, Pittsburgh, PA) after hand pulling glass needles from the tips of the rods using a butane torch.

## Supporting Information

Figure S1 Multiple sequence alignment of the *Stentor* argonaute homologs with the canonical “DDH” motif and human PIWIL1; important residues are highlighted in red. The alignment was performed using ClustalW2.
(TIF)

Figure S2 RNAi knockdown of α- or β-tubulin yields similar morphological defects. (A) Immunofluorescence image of a stained control cell; cortical rows (green, anti–acetylated-tubulin) and macronucleus (blue, DAPI). (B) Immunofluorescence image of a stained *α-tubulin(RNAi)* cell; cortical rows (green, anti–acetylated-tubulin) and macronucleus (blue, DAPI). (C) Brightfield images of cells fed either control, split β-tubulin, or α-tubulin vectors. (D) Brightfield image of an *α-tubulin(RNAi)* cell that developed an ectopic posterior pole (arrow).
(TIF)

Figure S3 Control RNAi cells have normal morphologies. (A) Brightfield image of an *LF4(RNAi)* cell. LF4 is a kinase involved in ciliary length control but not expected to play any role in cortical patterning. We identified 24 potential LF4 homologs from the PRICE assembly using reciprocal-best-BLAST hits and cloned the top hit. As expected, cell shape was completely normal in the *LF4(RNAi)* cells. (B, C) DIC images of control and *LF4(RNAi)* cells' posterior region showing their cilia; image taken at 40×. *LF4(RNAi)* cells have significantly longer cilia (arrows), confirming that RNAi of LF4 was effective. (D) Graph of cilia lengths for control and *LF4(RNAi)* cells after 3 and 4 d of feeding the RNAi vectors. (E, F) Brightfield images of both planarian *ODF2(RNAi)* and *C. elegans unc22(RNAi)*, genes not present in *Stentor*, which have no obvious phenotype.
(TIF)

Figure S4 Immunofluorescence images showing that the signal in the posterior disappears when the anti-Mob1 antibody is pre-incubated with the immunizing peptide before staining. Under these conditions, punctate staining in the nucleus dominates, suggesting that it is off-target or nonspecific staining.
(TIF)

Figure S5 Clustal Omega alignment of all Stentor Mob1 nucleotide sequences was performed using default settings. Using Mob1a as a reference point for pairwise alignments to all other Mob1 homologs, there is at least one 20 mer predicted to be shared between each of the pairs.
(TIF)

Figure S6 Cell shape analysis of control and *Mob1(RNAi)* cells shows a loss of normal proportions. (A) Brightfield images used as the input for the cell shape analysis process. (B) Thresholded black and white images are then further processed by two rounds of the smoothing function in ImageJ. (C) The cell outline was detected using the black and white image as an input for our MatLab program, and the image was skeletonized to find the midline. (D) The cell midline is then fit to a curve and perpendicular lines are drawn. The intersection of these perpendicular lines with the cell outlines (red and pink X's) are then used to determine the cell widths.
(TIF)

Figure S7 Brightfield images of split *Mob1(RNAi)* constructs. Targeting either the first half (40–356) or the second half (326–672) yielded the elongated cell phenotype after 5 d of feeding.
(TIF)

Figure S8 Brightfield images of control and *Mob1(RNAi)* cells at the end of cell division. Control cells separate properly at the end of cytokinesis, whereas the *Mob1(RNAi)* cells remain attached.
(TIF)

Figure S9 Graph displaying data from a population of 50 cells fed the Mob1 RNAi vector over the course of 172 h. Cells were visually scored for phenotypes once per day for either a normal, elongated, or medusoid appearance. Any increase in the total number of cells above 50 is the result of cell division during the course of the experiment. This experiment was done in triplicate, and the error bars represent the standard deviations for each time point.
(TIF)

Figure S10 Immunofluorescence image showing a cell 3 h after the surgical removal of both the anterior and posterior. Mob1 (green, anti-Mob1) can be seen in the posterior of the cell, indicated by the white arrowhead.
(TIF)

Movie S1 Brightfield microscopy of *Stentor* cells fed the full-length Mob1_(40–672)_ vector for 5 d. Cells were imaged on a stereoscope in a nine-well spot plate. At this stage all of the cells have progressed to the medusoid phenotype.
(MOV)

Movie S2 Brightfield microscopy of *Stentor* cells fed the Mob1_(40–356)_ vector for 5 d. Cells were imaged on a stereoscope in a nine-well spot plate. Cells have still progressed to the elongated phenotype.
(MOV)

Movie S3 Brightfield microscopy of *Stentor* cells fed the Mob1_(326–672)_ vector for 5 d. Cells were imaged on a stereoscope in a nine-well spot plate. Cells have still progressed to the elongated phenotype.
(MOV)

Table S1 List of identified RNAi machinery homologs in *Stentor*. Argonaute (Sciwi), Dicer, and RNA-dependant RNA polymerases were identified from genomic sequence using a best-reciprocal BLAST approach starting with annotated *Paramecium tetraurelia* proteins. For each gene the Genbank accession number and the top hit in *Paramecium* is also shown with its corresponding E-value.
(XLSX)

Table S2 List of genes used in the phylogenetic analysis of the argonaute homologs. Each gene has the species listed along with its gene ID.
(XLSX)

Table S3 Summary of all RNAi constructs used in this experiment, their lengths and relative positions within the coding sequence, and primers used to amplify the sequences from genomic samples.
(XLSX)

Table S4 Oligonucleotide sequences used for the qRT-PCR analysis of *Stentor* genes.
(XLSX)

## Abbreviations

OA: oral apparatus
RNAi: RNA interference
