# Supplementary figures and images for: The Kinase Regulator Mob1 Acts as a Patterning Protein for Stentor Morphogenesis
Source: PLoS Biol. 2014 May 13;12(5):e1001861. doi: 10.1371/journal.pbio.1001861 (PMC4019465; doi:10.1371/journal.pbio.1001861)

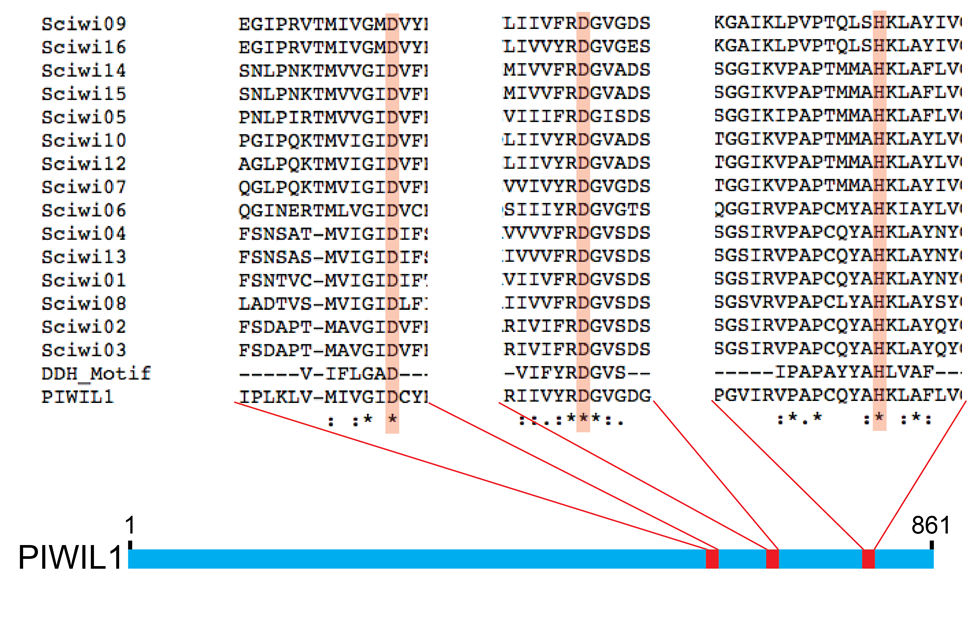

Supplement: Figure S1 — Multiple sequence alignment of the Stentor argonaute homologs with the canonical “DDH” motif and human PIWIL1; important residues are highlighted in red. The alignment was performed using ClustalW2. (TIF) [file pbio.1001861.s001.tif]

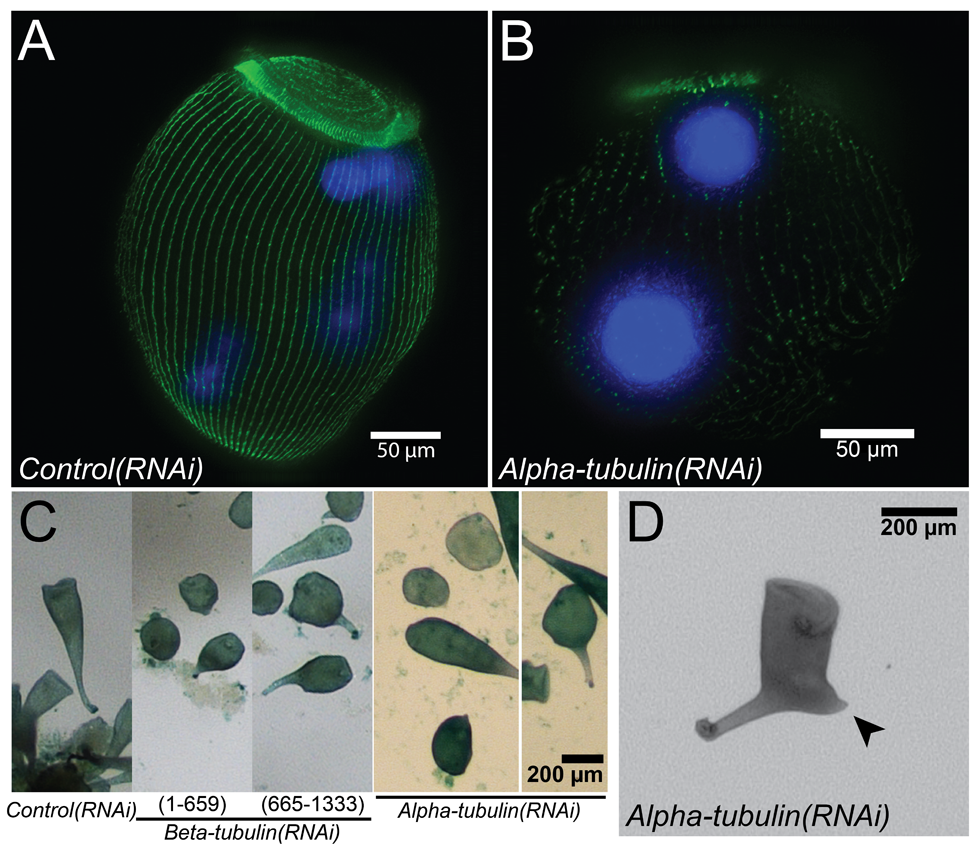

Supplement: Figure S2 — RNAi knockdown of α- or β-tubulin yields similar morphological defects. (A) Immunofluorescence image of a stained control cell; cortical rows (green, anti–acetylated-tubulin) and macronucleus (blue, DAPI). (B) Immunofluorescence image of a stained α-tubulin(RNAi) cell; cortical rows (green, anti–acetylated-tubulin) and macronucleus (blue, DAPI). (C) Brightfield images of cells fed either control, split β-tubulin, or α-tubulin vectors. (D) Brightfield image of an α-tubulin(RNAi) cell that developed an ectopic posterior pole (arrow). (TIF) [file pbio.1001861.s002.tif]

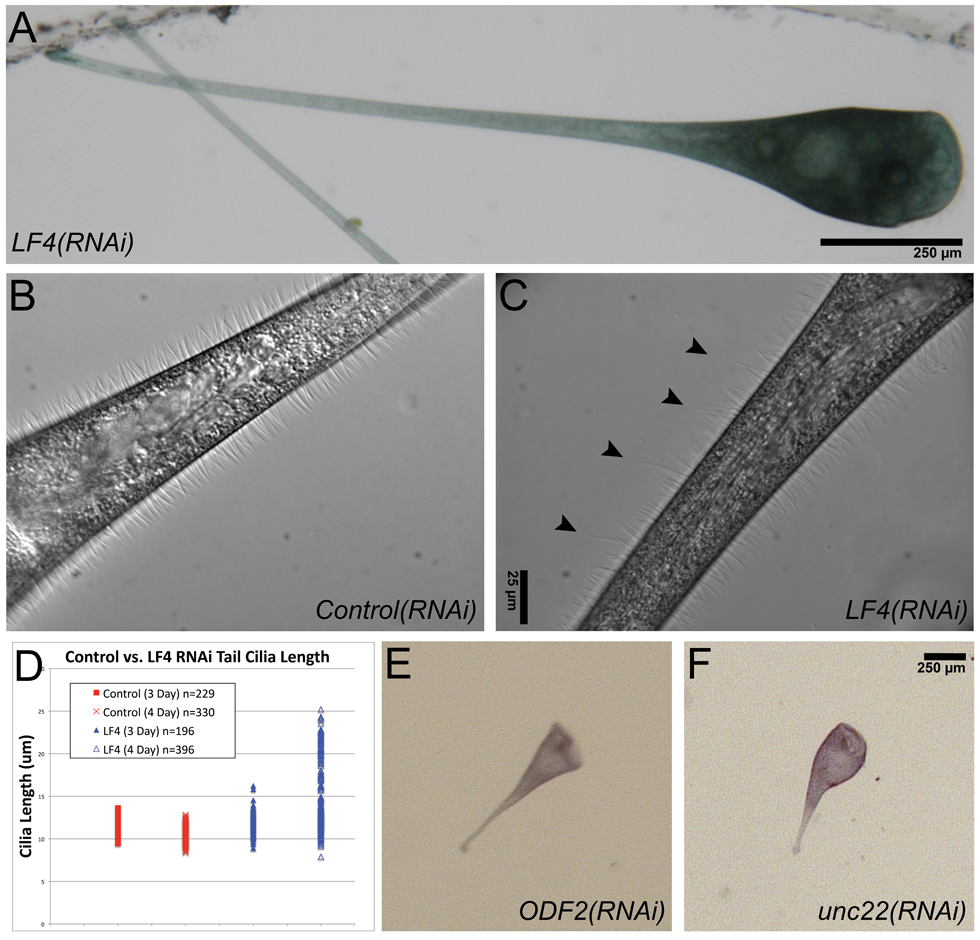

Supplement: Figure S3 — Control RNAi cells have normal morphologies. (A) Brightfield image of an LF4(RNAi) cell. LF4 is a kinase involved in ciliary length control but not expected to play any role in cortical patterning. We identified 24 potential LF4 homologs from the PRICE assembly using reciprocal-best-BLAST hits and cloned the top hit. As expected, cell shape was completely normal in the LF4(RNAi) cells. (B, C) DIC images of control and LF4(RNAi) cells' posterior region showing their cilia; image taken at 40×. LF4(RNAi) cells have significantly longer cilia (arrows), confirming that RNAi of LF4 was effective. (D) Graph of cilia lengths for control and LF4(RNAi) cells after 3 and 4 d of feeding the RNAi vectors. (E, F) Brightfield images of both planarian ODF2(RNAi) and C. elegans unc22(RNAi), genes not present in Stentor, which have no obvious phenotype. (TIF) [file pbio.1001861.s003.tif]

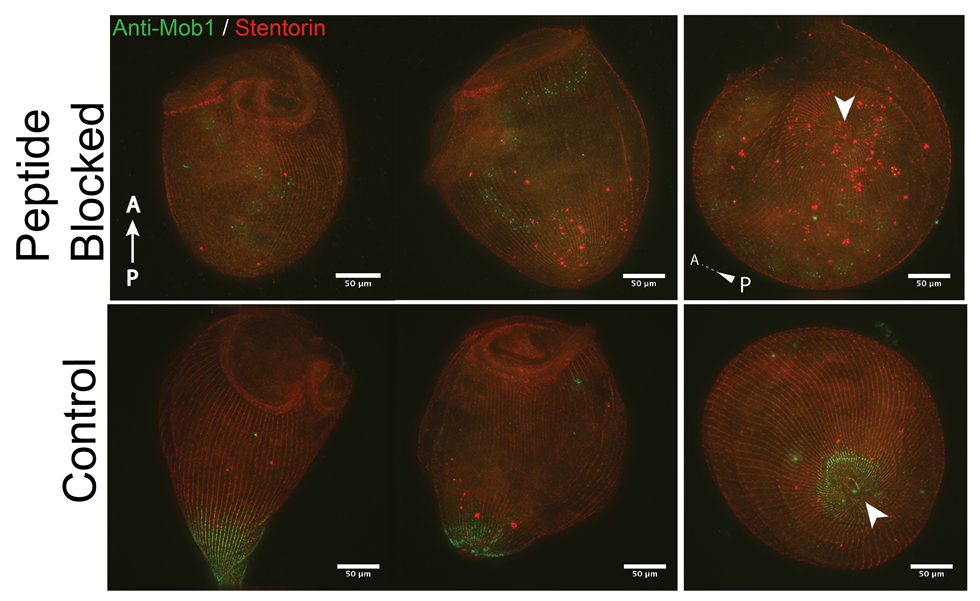

Supplement: Figure S4 — Immunofluorescence images showing that the signal in the posterior disappears when the anti-Mob1 antibody is pre-incubated with the immunizing peptide before staining. Under these conditions, punctate staining in the nucleus dominates, suggesting that it is off-target or nonspecific staining. (TIF) [file pbio.1001861.s004.tif]

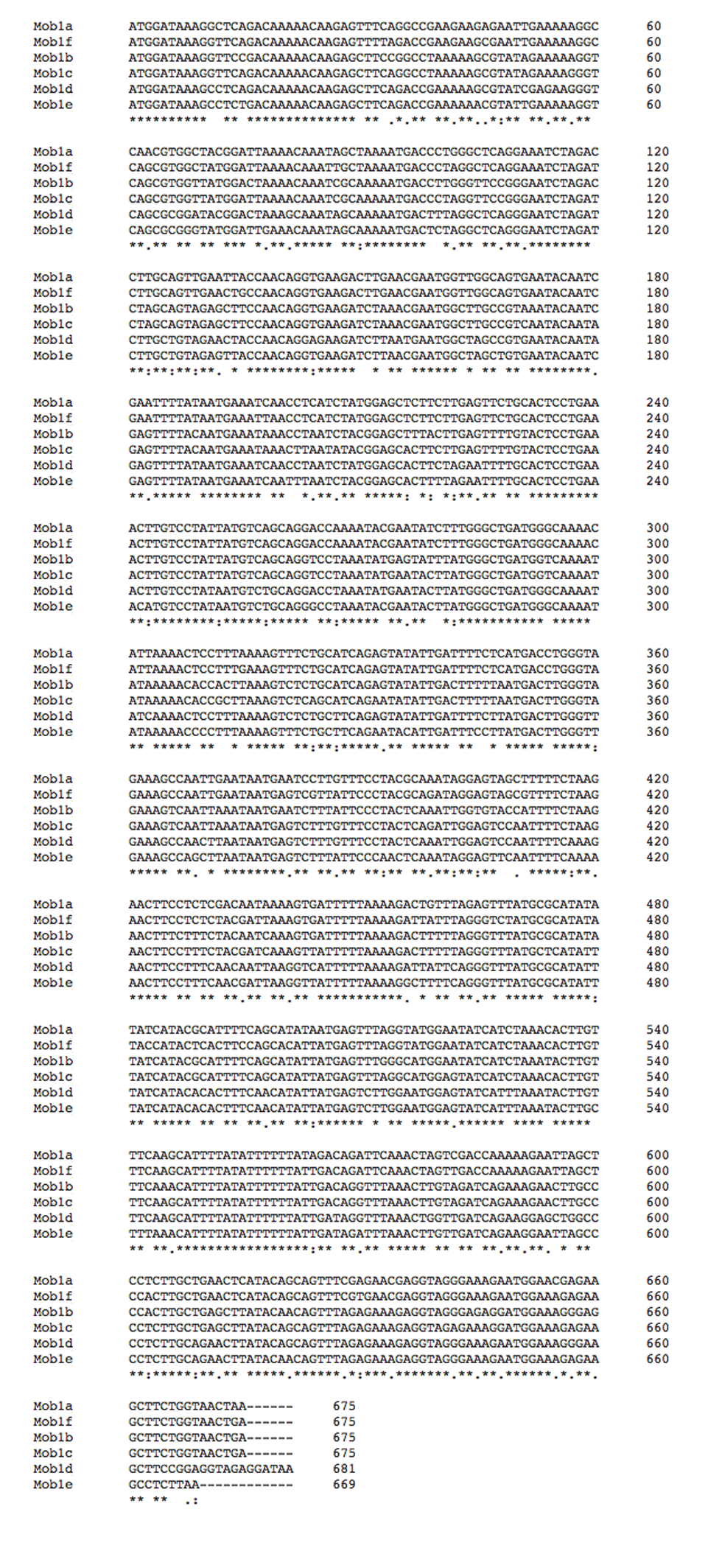

Supplement: Figure S5 — Clustal Omega alignment of all Stentor Mob1 nucleotide sequences was performed using default settings. Using Mob1a as a reference point for pairwise alignments to all other Mob1 homologs, there is at least one 20 mer predicted to be shared between each of the pairs. (TIF) [file pbio.1001861.s005.tif]

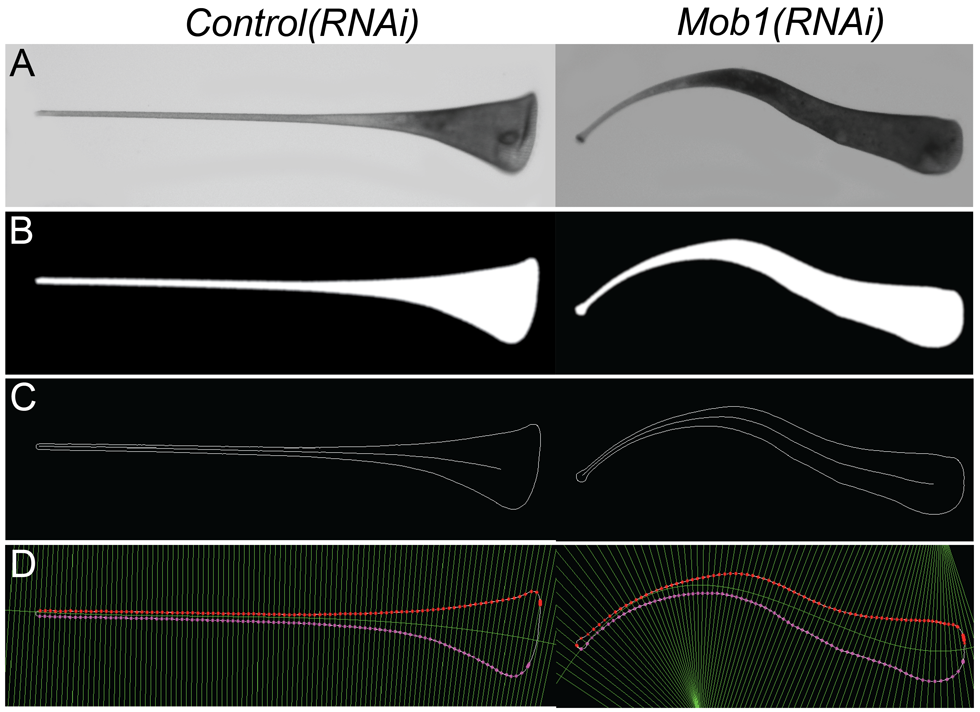

Supplement: Figure S6 — Cell shape analysis of control and Mob1(RNAi) cells shows a loss of normal proportions. (A) Brightfield images used as the input for the cell shape analysis process. (B) Thresholded black and white images are then further processed by two rounds of the smoothing function in ImageJ. (C) The cell outline was detected using the black and white image as an input for our MatLab program, and the image was skeletonized to find the midline. (D) The cell midline is then fit to a curve and perpendicular lines are drawn. The intersection of these perpendicular lines with the cell outlines (red and pink X's) are then used to determine the cell widths. (TIF) [file pbio.1001861.s006.tif]

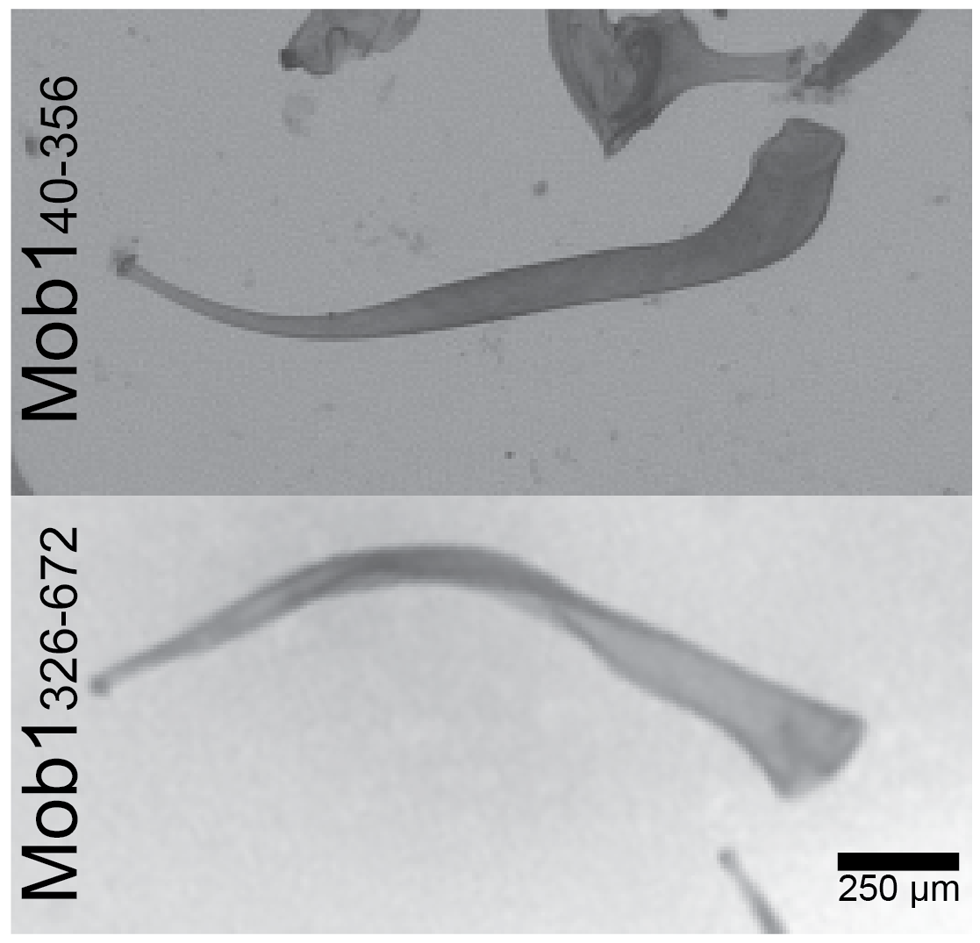

Supplement: Figure S7 — Brightfield images of split Mob1(RNAi) constructs. Targeting either the first half (40–356) or the second half (326–672) yielded the elongated cell phenotype after 5 d of feeding. (TIF) [file pbio.1001861.s007.tif]

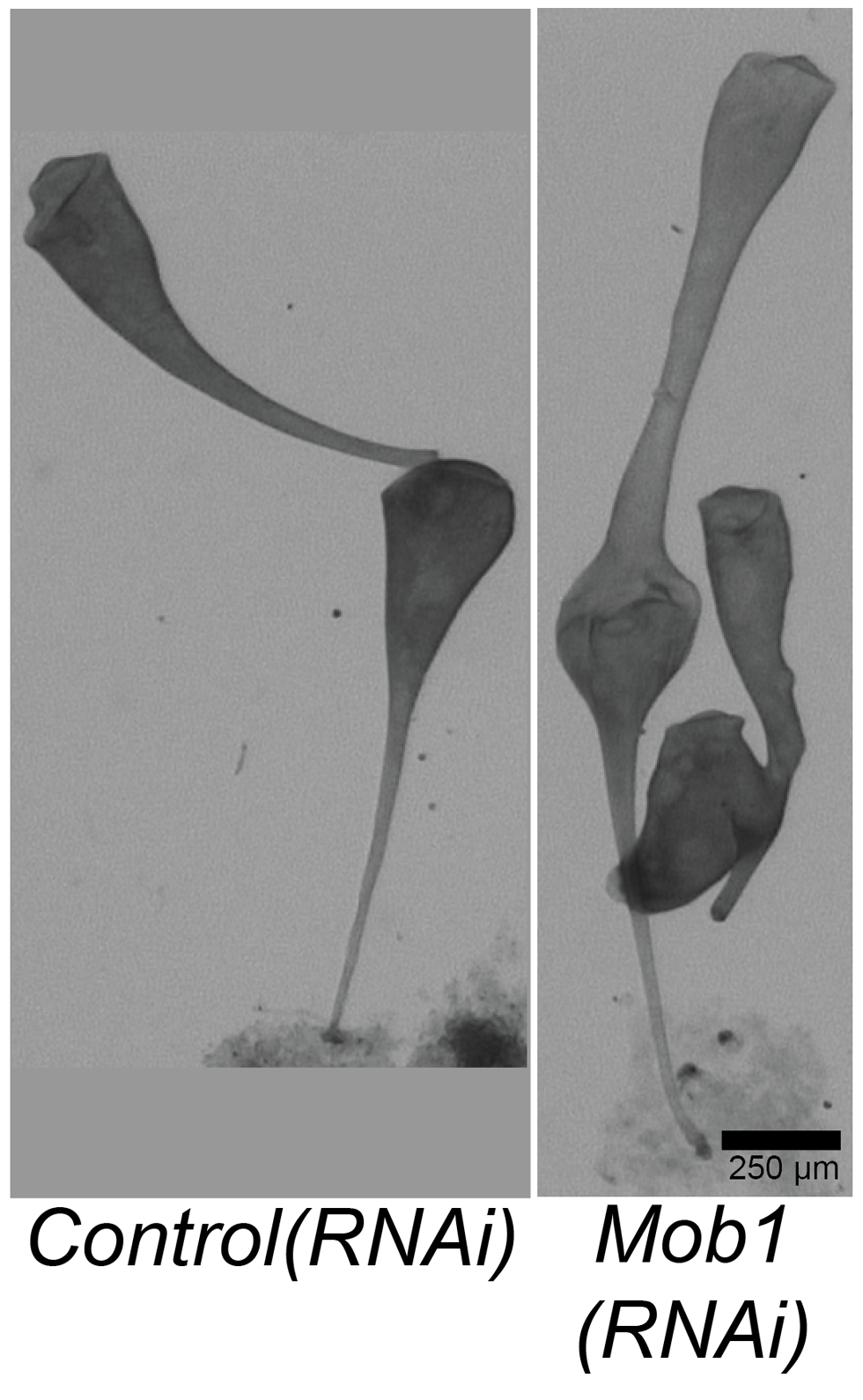

Supplement: Figure S8 — Brightfield images of control and Mob1(RNAi) cells at the end of cell division. Control cells separate properly at the end of cytokinesis, whereas the Mob1(RNAi) cells remain attached. (TIF) [file pbio.1001861.s008.tif]

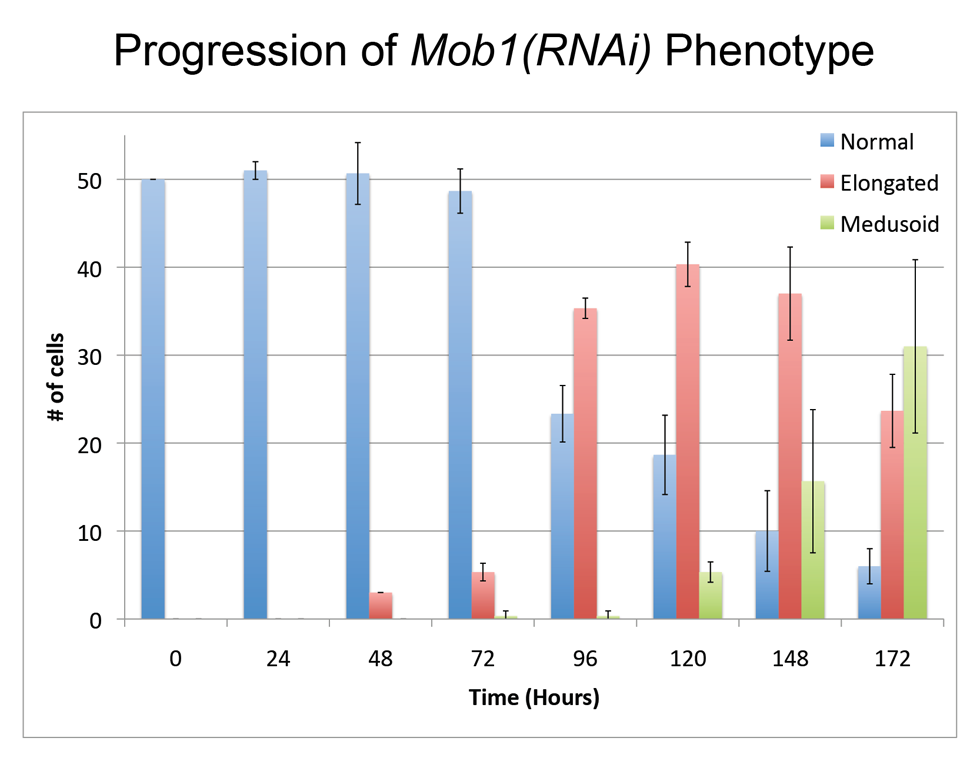

Supplement: Figure S9 — Graph displaying data from a population of 50 cells fed the Mob1 RNAi vector over the course of 172 h. Cells were visually scored for phenotypes once per day for either a normal, elongated, or medusoid appearance. Any increase in the total number of cells above 50 is the result of cell division during the course of the experiment. This experiment was done in triplicate, and the error bars represent the standard deviations for each time point. (TIF) [file pbio.1001861.s009.tif]

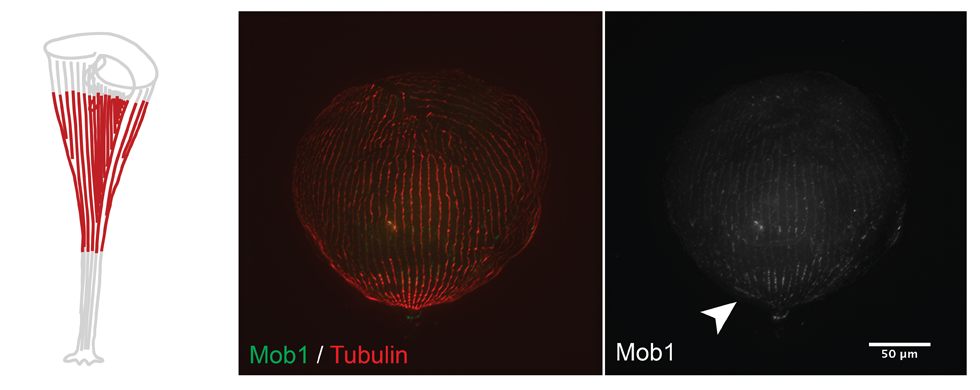

Supplement: Figure S10 — Immunofluorescence image showing a cell 3 h after the surgical removal of both the anterior and posterior. Mob1 (green, anti-Mob1) can be seen in the posterior of the cell, indicated by the white arrowhead. (TIF) [file pbio.1001861.s010.tif]
